# The sole introduction of two single-point mutations establishes glycerol utilization in *Saccharomyces cerevisiae* CEN.PK derivatives

**DOI:** 10.1186/s13068-016-0696-6

**Published:** 2017-01-03

**Authors:** Ping-Wei Ho, Steve Swinnen, Jorge Duitama, Elke Nevoigt

**Affiliations:** 1 Department of Life Sciences and Chemistry, Jacobs University Bremen gGmbH, Campus Ring 1, 28759 Bremen, Germany; 2 Systems and Computing Engineering Department, Universidad de los Andes, Cra 1 Este No 19A-40, Bogotá, Colombia

**Keywords:** Yeast, CEN.PK, Glycerol, Adaptive laboratory evolution, Evolutionary engineering

## Abstract

**Background:**

Glycerol is an abundant by-product of biodiesel production and has several advantages as a substrate in biotechnological applications. Unfortunately, the popular production host *Saccharomyces cerevisiae* can barely metabolize glycerol by nature.

**Results:**

In this study, two evolved derivatives of the strain CEN.PK113-1A were created that were able to grow in synthetic glycerol medium (strains PW-1 and PW-2). Their growth performances on glycerol were compared with that of the previously published evolved CEN.PK113-7D derivative JL1. As JL1 showed a higher maximum specific growth rate on glycerol (0.164 h^−1^ compared to 0.119 h^−1^ for PW-1 and 0.127 h^−1^ for PW-2), its genomic DNA was subjected to whole-genome resequencing. Two point mutations in the coding sequences of the genes *UBR2* and *GUT1* were identified to be crucial for growth in synthetic glycerol medium and subsequently verified by reverse engineering of the wild-type strain CEN.PK113-7D. The growth rate of the resulting reverse-engineered strain was 0.130 h^−1^. Sanger sequencing of the *GUT1* and *UBR2* alleles of the above-mentioned evolved strains PW-1 and PW-2 also revealed one single-point mutation in these two genes, and both mutations were demonstrated to be also crucial and sufficient for obtaining a maximum specific growth rate on glycerol of ~0.120 h^−1^.

**Conclusions:**

The current work confirmed the importance of *UBR2* and *GUT1* as targets for establishing glycerol utilization in strains of the CEN.PK family. In addition, it shows that a growth rate on glycerol of 0.130 h^−1^ can be established in reverse-engineered CEN.PK strains by solely replacing a single amino acid in the coding sequences of both Ubr2 and Gut1.

**Electronic supplementary material:**

The online version of this article (doi:10.1186/s13068-016-0696-6) contains supplementary material, which is available to authorized users.

## Background

Due to the vast surplus of crude glycerol accompanying the thriving biodiesel industry, glycerol has become an attractive substrate for biotechnological processes [1–3]. There are several reasons why glycerol can be an interesting alternative to sugars as a source of carbon and energy. One reason is that glycerol is more reduced than sugars, providing more reducing power for the production of small molecules with a high degree of reduction [4]. Another reason is that glycerol is metabolized in a fully respiratory manner in most microorganisms including the popular yeast *Saccharomyces cerevisiae.* This implies that the use of glycerol does not exert a Crabtree effect in *S. cerevisiae*, thereby circumventing the need to precisely control the sugar concentration in processes aiming at the production of biomass and growth-related molecules [5].

*Saccharomyces cerevisiae* is an attractive production organism in industrial biotechnology, and the palette of compounds currently produced by this organism by far exceeds the traditionally known products bioethanol and carbon dioxide. The preferred status of *S. cerevisiae* in industrial biotechnology is the result of the enormous body of knowledge available on its physiology and genetics, the intense experience with this organism in industrial bioprocesses, its robustness under process conditions, and the ease to genetically engineer the organism [6]. In particular, strains of the CEN.PK family have become a popular platform in both fundamental research and industrial applications as they offer a good compromise between physiological properties (e.g., the growth characteristics in shake-flask cultures) and genetic properties (e.g., transformation efficiency) [7]. CEN.PK strains have been applied in numerous metabolic and evolutionary engineering studies, such as for the production of lactate and pyruvate [8, 9], isoprenoids [10, 11], C_4_-dicarboxylic acids [12], ornithine [13], n-butanol [14], and 3-hydroxypropionic acid [15], and for the fermentation of pentose sugars [16–18]. Although some of these processes could profit from utilizing glycerol as a carbon source due to its high reducing power, wild-type strains of the CEN.PK family cannot utilize glycerol at all in synthetic medium, particularly when complex medium supplements such as yeast extract or peptone are omitted [19, 20].

A previous study conducted in our laboratory showed that certain *S.* *cerevisiae* strains are able to grow in synthetic glycerol medium with maximum specific growth rates (µ_max_) up to 0.15 h^−1^ [20], confirming the previously reported high intraspecies diversity with regard to this phenotype [21]. The polygenetic basis of the glycerol growth phenotype in a haploid meiotic segregant (CBS 6412-13A) from the glycerol-utilizing isolate CBS 6412 in comparison to the non-growing, well-established laboratory strain CEN.PK113-1A was recently unraveled, and the knowledge was used for reverse engineering of the latter strain [22]. The identified CBS allele *UBR2*_CBS_ showed the highest impact on glycerol growth performance in strain CEN.PK113-1A after allele replacement, resulting in a µ_max_ on glycerol of 0.04 h^−1^. An additional positive effect was obtained by also replacing the *GUT1*_CBS_ allele (µ_max_ of 0.08 h^−1^) and the *SSK1*_CBS_ allele (µ_max_ of 0.09 h^−1^) in the strain CEN.PK113-1A *UBR2*_CBS_. The growth rate of the final reverse-engineered strain was 68% of the rate obtained for the wild-type CBS 6412-13A strain (µ_max_ of 0.13 h^−1^) [22].

The results obtained by the above-described reverse engineering approach have been very promising and raised the question whether and how even higher maximum specific growth rates could be achieved in order to obtain strains attractive for real industrial applications. It is noteworthy in this context that several previous studies focusing on adaptive laboratory evolution (ALE) for improving growth on glycerol of *S. cerevisiae* strains have reported growth rates of evolved CEN.PK strains up to 0.22 h^−1^. ALE employs evolutionary mechanisms for improving a specific industrially relevant phenotype of a chosen microbial strain. Cells are subjected to precisely defined conditions for prolonged periods of time, allowing selection for improved phenotypes that have arisen by natural mutations occurring throughout the course of cultivation [23]. Using repeated batch cultivation on glycerol, Merico et al. [19] achieved a growth rate of 0.17 h^−1^ for the strain CEN.PK113-7D, while Ochoa-Estopier et al. [5] reported a growth rate of 0.20 h^−1^ for the same original strain and a growth rate of 0.22 h^−1^ for the industrial strain Ethanol Red. Although the evolved *S. cerevisiae* strains show even higher growth rates on glycerol than the above-mentioned reverse-engineered derivatives using the superior alleles of the CBS 6412-13A wild-type isolate [22], the mutations underlying the glycerol growth phenotype of the evolved strains have not been reported so far.

The goal of the current study was to find additional or superior genetic targets for reverse engineering of CEN.PK strains by scrutinizing the underlying genetic basis of the improved glycerol growth phenotype in evolved CEN.PK derivatives. The first part of the work focuses on generating CEN.PK113-1A derivatives with improved growth in synthetic glycerol medium via ALE and comparing their growth performance with that of the strain JL1, a derivative of the strain CEN.PK113-7D that was previously evolved for glycerol utilization by Ochoa-Estopier et al. [5]. The second part concerns the identification of the crucial mutations in the evolved strains and the reverse engineering of a CEN.PK wild-type strain.

## Methods

### Strain and medium composition

All *S. cerevisiae* strains used in this study are listed in Table 1. Yeast cells were routinely cultivated in a static incubator at 30 °C and maintained on solid YPD medium containing 1% (w/v) yeast extract, 2% (w/v) peptone, 2% (w/v) glucose, and 1.5% (w/v) agar. The selection of strains transformed with the *ble* ^*r*^ selectable genetic marker was conducted in solid YPD medium supplemented with 20 µg mL^−1^ phleomycin. The induction of *HO* gene was carried out in liquid YPGal medium containing 1% (w/v) yeast extract, 2% (w/v) peptone, and 2% (w/v) galactose. Experiments for assaying yeast growth and evolution were performed in synthetic medium according to Verduyn et al. [24] containing per liter: 5 g (NH_4_)_2_SO_4_, 3 g KH_2_PO_4_, 0.5 g MgSO_4_·7H_2_O, 15 mg EDTA, 4.5 mg ZnSO_4_.·7H_2_O, 0.84 mg MnCl_2_·2H_2_O, 0.3 mg CoCl_2_·6H_2_O, 0.3 mg CuSO_4_·5H_2_O, 0.4 mg NaMoO_4_·2H_2_O, 4.5 mg CaCl_2_·2H_2_O, 3 mg FeSO_4_·7H_2_O, 1 mg H_3_BO_3_, and 0.1 mg KI. Filter sterilized vitamins were added after heat sterilization of this medium. The final amounts of vitamins per liter medium were 0.05 mg D-(+)-biotin, 1 mg D-pantothenic acid hemicalcium salt, 1 mg nicotinic acid, 25 mg myo-inositol, 1 mg thiamine chloride hydrochloride, 1 mg pyridoxine hydrochloride, and 0.2 mg 4-aminobenzoic acid. The carbon source added to the medium was either 2% (w/v) glucose or 6% (v/v) glycerol. The pH was adjusted to 6.5 with 2 M KOH for the synthetic glucose medium, and to 4.0 with 2 M H_3_PO_4_ for the synthetic glycerol medium. For the preparation of solid synthetic galactose medium, 20 g L^−1^ agar was first washed three times with deionized water prior to adding it to the medium before autoclaving. The washing step is necessary to reduce the background growth of *S. cerevisiae* observed on solid synthetic medium without adding any carbon source. The selection of strains for loss of the *GIN11* counter-selectable marker was carried out in solid synthetic medium with 2% (w/v) galactose instead of glucose. The backcrossing studies were carried out on solid YPD medium. For sporulation of diploid strains, cells were grown on solid sporulation medium containing 1% (w/v) potassium acetate, 0.05% (w/v) potassium bicarbonate, and 1.5% (w/v) agar with pH adjusted to 6.0 [25].

**Table 1:** Yeast strains used in this study

Strain	Genotype, description	Reference
CEN.PK113-1A	*MAT*α (prototrophic)	Euroscarf
PW-1 (CEN.PK113-1A)	*MAT*α (prototrophic) evolved for growth on glycerol	This study
PW-2 (CEN.PK113-1A)	*MAT*α (prototrophic) evolved for growth on glycerol	This study
CBS 6412-13A	*MAT*a haploid segregant of CBS 6412	[20]
CEN.PK113-1A *GUT1*_CBS_ *UBR2*_CBS_ *SSK1*_CBS_	*MAT*α; *gut1*::*GUT1*_*CBS 6412*-*13A*_ *ubr2*::*UBR2*_*CBS 6412*-*13A*_ *ssk1*::*SSK1*_*CBS 6412*-*13A*_	[22]
CEN.PK113-7D	*MAT*a (prototrophic)	[5]
JL1 (CEN.PK113-7D)	*MAT*a (prototrophic); evolved for growth on glycerol	[5]
JL1-2 (CEN.PK113-7D)	*MAT*α (prototrophic); mating type switched from JL1	This study
CEN.PK113-7D *GUT1*_JL1_	*MAT*a; *gut1*::*GUT1*_*JL1*_ allelic replacement analysis of *GUT1*_*JL1*_	This study
CEN.PK113-7D *GUT1*_PW-1_	*MAT*a; *gut1*::*GUT1*_*PW*-*1*_ allelic replacement analysis of *GUT1*_*PW*-*1*_	This study
CEN.PK113-7D *GUT1*_PW-2_	*MAT*a; *gut1*::*GUT1*_*PW*-*2*_ allelic replacement analysis of *GUT1*_*PW*-*2*_	This study
CEN.PK113-7D *GUT1*_CBS_	*MAT*a; *gut1*::*GUT1*_*CBS*_ allelic replacement analysis of *GUT1*_*CBS 6412*-*13A*_	This study
CEN.PK113-7D *UBR2*_JL1_	*MAT*a; *ubr2*::*UBR2*_*JL1*_ allelic replacement analysis of *UBR2*_*JL1*_	This study
CEN.PK113-7D *UBR2*_PW-1_	*MAT*a; *ubr2*::*UBR2*_*PW*-*1*_ allelic replacement analysis of *UBR2*_*PW*-*1*_	This study
CEN.PK113-7D *UBR2*_PW-2_	*MAT*a; *ubr2*::*UBR2*_*PW*-*2*_ allelic replacement analysis of *UBR2*_*PW*-*2*_	This study
CEN.PK113-7D *UBR2*_CBS_	*MAT*a; *ubr2*::*UBR2*_*CBS*_ allelic replacement analysis of *UBR2*_*CBS 6412*-*13A*_	This study
CEN.PK113-7D *GUT1*_JL1_ *UBR2*_JL1_	*MAT*a; *gut1*::*GUT1*_*JL1*_ *ubr2*::*UBR2*_*JL1*_ allelic replacement analysis of *GUT1*_*JL1*_ and *UBR2*_*JL1*_	This study
CEN.PK113-7D *GUT1*_PW-1_ *UBR2*_PW-1_	*MAT*a; *gut1*::*GUT1*_*PW*-*1*_ *ubr2*::*UBR2*_*PW*-*1*_ allelic replacement analysis of *GUT1*_*PW*-*1*_ and *UBR2*_*PW*-*1*_	This study
CEN.PK113-7D *GUT1*_PW-2_ *UBR2*_PW-2_	*MAT*a; *gut1*::*GUT1*_*PW*-*2*_ *ubr2*::*UBR2*_*PW*-*2*_ allelic replacement analysis of *GUT1*_*PW*-*2*_ and *UBR2*_*PW*-*2*_	This study
CEN.PK113-7D *GUT1*_CBS_ *UBR2*_CBS_	*MAT*a; *gut1*::*GUT1*_*CBS*_ *ubr2*::*UBR2*_*CBS*_ allelic replacement analysis of *GUT1*_*CBS 6412*-*13A*_ and *UBR2*_*CBS 6412*-*13A*_	This study

### Serial transfer for laboratory adaptive evolution

A preculture of strain CEN.PK113-1A was prepared in synthetic medium containing 6% (v/v) glycerol supplemented with 0.77 g/L CSM-URA (Qbiogene, USA) and 0.15 g/L uracil at 30 °C on a rotary shaker (200 rpm) overnight. The addition of CSM resulted in the following supplementation (per L): 10 mg adenine, 50 mg l-arginine HCl, 80 mg l-aspartic acid, 20 mg l-histidine HCl, 50 mg l-isoleucine, 100 mg l-leucine, 50 mg l-lysine HCl, 20 mg l-methionine, 50 mg l-phenylalanine, 100 mg l-threonine, 50 mg l-tryptophan, 50 mg tyrosine, 140 mg l-valine, and 150 mg uracil. This culture was then used as the inoculum for both of the sequential batch cultures. The first evolution line was carried out in fresh synthetic glycerol medium in which the CSM concentration was gradually reduced by 10% in each subculture, i.e., only 90% of the CSM concentration present in the preculture was added to the second batch, 80% to the third batch, and so forth until the 10th serial transfers where 10% of the original CSM supplementation was last added. All subsequent subcultivations were carried out in synthetic glycerol medium without supplementation. The second evolution line was carried out in synthetic glycerol medium without any addition of CSM. During both evolution lines, cells were harvested from the mid-exponential phase and reinoculated the following subcultivation with an OD_600_ adjusted to 0.2 (corresponds to 4.0 × 10^6^ cells per mL). After approximately 55 generations, a colony from each evolution line was isolated and subjected to the quantitative analysis of growth in liquid glycerol medium. Serial subcultivations of the evolved isolates and JL1 in glucose medium were carried out by transferring the cells into fresh synthetic glucose medium adjusting an OD_600_. In total, 5 subcultivations (corresponding to ca. 25 generations) were conducted in shake flasks.

### Mating type switch, mating, sporulation, and tetrad analysis

Mating type of the haploid strains was switched by inducing transient expression of the *HO* gene, an endonuclease which specifically cleaves at the *MAT* locus, leading to the switch of mating type via the subsequent repairing mechanism of the damaged DNA [26]. In more detail, cells were transformed with plasmid pFL39GAL1HO [27], on which *HO* gene is controlled by the galactose-inducible promoter *GAL1* and then grown in YPGal medium to induce the expression of *HO* gene. After 2 h of incubation, aliquots of the cell suspension were streaked on solid YPD medium every hour. The correct mating type was verified by diagnostic PCR of the *MAT* locus using *MAT*-*ver* primers [28] (Table 2). Haploid strains with desired mating type were subsequently cultivated for several generations in liquid YPD medium for the loss of plasmid. Mating, sporulation, and tetrad analysis were performed by procedures described by Sherman and Hicks [25]. Tetrads were dissected by using the micromanipulator from Singer Instruments (Roadwater Watchet Somerset, UK).

**Table 2:** Primers used in this study

Primer name	Sequence
*MAT*-*fwd*	AGTCACATCAAGATCGTTTATGG
*MATa*-*rv*	ACTCCACTTCAAGTAAGAGTTTG
*MATα*-*rv*	GCACGGAATATGGGACTACTTCG
*ble*-*fw*	AGATCTGTTTAGCTTGCCTCG
*GIN11*-*rv*	CGAGGCAAGCTAAACAGATCTTCCAAGCTTGGGATCCGG
*UBR2*-*GIN11*-*fw*	AACGAGTAAACCACCACTTCGCTCGAATCGCCAAGCTTTCTAGATGTACACCCACGCTGTATCGGAACCCTAAAGGGAGC
*UBR2*-*ble*-*rv*	CGGTGTTGAACGCAAGCTATCTACCTCTTTCTTTGATGCGGTTGAAATATTATACTCGAAGCATAGGCCACTAGTGGATCTG
*UBR2*-*control*-*fw*	CTTATCTGCGTTGCAGCTTC
*UBR2*-*control*-*rv*	CATCGTTGTCGTTACTCTGC
*GUT1*-*GIN11*-*fw*	CGAACCATATAAAATATACCATGTGGTTTGAGTTGTGGCCGGAACTATACAAATAGTTATATCGGAACCCTAAAGGGAGC
*GUT1*-*ble*-*rv*	AAGGTGGAGAGGAATATAAAATTATGGAAATTACATTGTTAATAGAAATTATTTATGTTGGCATAGGCCACTAGTGGATCTG
*GUT1*-*control*-*fw*	GACATGTGTAACCTCTCGAC
*GUT1*-*control*-*rv*	TCAAGGCAACAACGTGGTTC

### Whole-genome resequencing analysis

Strains chosen for whole-genome resequencing were cultivated individually in 50 mL of YPD medium at 30 °C with orbital shaking at 200 rpm for 16 h. Genomic DNA was extracted according to Johnston [29]. At least 5 μg of each genomic DNA sample was provided to BGI Tech Solutions (strain JL1) and to GATC Biotech AG (strain CEN.PK113-7D and F3) for sequence analysis using the Illumina HiSeq 2000 and 2500 platform, respectively. Paired-end reads of ~500 bp were generated for JL1 (17.3 M reads) and of ~100 bp for F3 (11.9 M reads) and CEN.PK113-7D (10.3 M reads). The average sequencing depths of the samples were 157, 95, and 85, respectively. Whole-genome resequencing data can be accessed at the SRA [30], accession number SRP093879.

Bioinformatic analysis of whole-genome resequencing data was performed following the NGSEP pipeline v2.1.5 [31] with the default parameters set on its graphical interface, except for the ploidy which was set to 1 and the minimum genotype quality score set to 60. In brief, the genotype quality score (GQ field in the VCF format) indicates posterior probability to discover a novel variant against the reference or to genotype an existing variant, encoded as a Phred Score (0–255). Larger values for the minimum threshold not only reduce the error rate but also reduce the number of variants discovered or genotyped, while smaller values not only increase the number of variants discovered or genotyped but also increase the error rate. The large sequencing depth at which the samples were sequenced allowed increasing the minimum quality score to 60, which theoretically means that one genotyping error is expected for each million data points. Details about the sensitivity and specificity of variants discovery and genotyping using different tools at different quality scores can be found at [31]. Regarding potential copy number variation (CNV) between the strains and S288C, both the CNVnator and the EWT algorithms were activated for the detection of CNVs based on the read depth signal. The VCF Annotation and Filtering modules of NGSEP were used to perform functional annotation of variants and to perform the following filters: (1) Retain only the variants in which the three samples showed a read depth greater than 20 reads and a genotype quality score greater than 60. (2) Retain only variants showing more than one allele within the sequenced samples (e.g., polymorphic within the three samples). (3) Remove variants with heterozygous genotype calls. (4) Remove variants spanning regions in which CNVs are predicted for the three samples. As a reference sequence, we used the latest version of the strain S288C reference genome available at the SGD website (www.yeastgenome.org/) and strain CEN.PK113-7D [32], respectively.

### Reverse engineering via allele replacement employing the GIN11 system

The endogenous *UBR2* or *GUT1* allele in the genome of strain CEN.PK113-7D was seamlessly replaced by the respective mutated version using the two-step *GIN11* counter-selectable system, respectively [33]. In the first step, a cassette harboring the *ble* ^*r*^ marker and a galactose-inducible growth inhibitor sequence (referred to as *GALp*-*GIN11M86*) was integrated into the CEN.PK113-7D genome at the target locus for the replacement of the *URR2* or *GUT1* allele. Next, the cassette was replaced by the respective mutated *GUT1* or *UBR2* allele amplified from genomic DNA of the relevant strains. The allele replacements were conducted based on the principle of in vivo homologous recombination in yeast. In more detail, the *ble* ^*r*^ marker and the *GALp*-*GIN11M86* sequence were amplified from pUG66 [34] and pGG119 [33], respectively, by PCR using the Phusion High-Fidelity DNA Polymerase (Thermo Fisher Scientific, USA) according to the manufacturer’s guidelines. Both the forward primer (*GENE*-*GIN11*-*fw*) used for obtaining the *GALp*-*GIN11M86* PCR product and the reverse primer (*GENE*-*ble*-*rv*) used for obtaining the *ble* ^*r*^ PCR product (Table 2) contained a 60-bp overhang at its 5′-terminus that is homologous to the sequence flanking the genomic region of the gene to be replaced, that is ca. 1000 bp upstream of the start codon and 300 bp downstream of the stop codon, respectively. Moreover, the reverse primer and the forward primer used for obtaining the *GALp*-*GIN11M86* and the *ble* ^*r*^ cassette were designed to generate a 40-bp complementary sequence between the two PCR products. After transforming the yeast cells with equimolar amounts of the two PCR products, the entire integration cassette (composed of *GALp*-*GIN11M86* and *ble* ^*r*^) was assembled at the respective target locus via homologous recombination. The transformants were selected on YPD medium containing 20 µg/mL phleomycin and verified by PCR with primers *ble*-*fw* and *GIN11*-*rv* (Table 2). Subsequently, the replacement of the *ble* ^*r*^/*GALp*-*GIN11M86* cassette in the generated *gut1* and *ubr2* deletion mutants was carried out by transforming them with the *GUT1* and/or *UBR2* mutant allele (replacement allele) from the strain of interest. The replacement alleles were obtained from the genomic DNA of the respective strains by PCR using Phusion High-Fidelity DNA Polymerase with primers *Allele*-*fw* and *Allele*-*rv* (Table 2), flanked by 60-bp sequences homologous to regions upstream and downstream of the chromosomal position of the cassette. Selection of transformants was achieved by plating the cells on solid synthetic medium with galactose for induction of the growth-inhibitory sequence *GIN11M86.* Correct integration of the replacement alleles was verified by diagnostic PCR using primers *Allele*-*control*-*fw* and *Allele*-*control*-*rv* (Table 2).

### Quantitative analysis of *S. cerevisiae* growth using the Growth Profiler

Quantitative analysis of *S. cerevisiae* growth on glycerol was carried out following the procedure described by Swinnen et al. [20]. The preculture was prepared by inoculating 4 mL of synthetic glucose medium with cells taken from a single cell colony on the YPD plate. The preculture was subsequently incubated overnight in an orbital shaker at 200 rpm at 30 °C. The preculture was used to inoculate 4 mL of fresh synthetic glucose medium adjusting an optical density (OD_600_) of 0.2. This culture (referred to as the intermediate culture hereafter) was subsequently grown for 48 h under the same conditions as the preculture. An appropriate amount of cells from the intermediate culture to obtain an OD_600_ of 0.2 (corresponds to 4.0 × 10^6^ cells per mL) in 5 mL medium was pelleted by centrifugation at 3000 rpm for 5 min, and washed once by resuspending the cells in 800 μL synthetic glycerol medium. After an additional centrifugation step, the cell pellet was resuspended in 5 mL of synthetic medium with glycerol. An aliquot of this final culture (750 μL each) was transferred into a well of a White Krystal™ 24-well clear bottom microplate (Porvair Sciences, United Kingdom) and cultivated in the Growth Profiler 1152 (Enzyscreen, The Netherlands) at 30 °C with orbital shaking at 200 rpm. The Growth Profiler was set to take a scan of the plate every 40 min. These scans were then used to calculate the density of the cultures expressed as green values (G-values). The G-values were subsequently converted to OD_600_ values (referred to here as OD_600_ equivalents) using a calibration curve: OD_600_ equivalent = 0.000000061761 * G-value^3.4784^.

### Determination of specific glycerol kinase activity

Yeast cell-free extract was prepared by harvesting about 2 × 10^8^ cells that were separated from culture supernatant by centrifugation at 4000 rpm and 4 °C for 10 min. Cells were washed twice in 5 mL of cold 50 mM potassium phosphate buffer (pH 7.5) containing 1 mM β-mercaptoethanol. The pellets were suspended in 1 mL of the same buffer with 1 g of acid-washed glass beads with a diameter of 425-600 µm (Sigma-Aldrich, USA). Cells were disrupted by vigorous vortexing for 15 min at 4 °C and the mixture was subsequently centrifuged for 5 min at 6000 rpm and 4 °C to remove glass beads and cell debris. A second centrifugation of the supernatant at 6000 rpm for 10 min at 4 °C was required to remove residual cell debris. The supernatant was directly used for the enzyme activity assays.

The glycerol kinase (GK) assay was adapted from the study of Hayashi and Lin [35] with few modifications. The phosphorylation of glycerol by GK is stimulated with the addition of ATP, and the release of ADP in turn can be followed spectrophotometrically by enzymatic coupling to NADH oxidation [36]. A complete assay mixture for one sample included the following (per mL): 10 µL of cell-free extract, 50 mM potassium phosphate at pH 7.5, 10 mM of MgCl_2_, 10 mM of glycerol, 1 mM of ATP, 1 mM of phosphoenolpyruvate, 0.5 mM of NADH, 0.066 mg of pyruvate kinase (Sigma-Aldrich, USA), and 0.033 mg of lactate dehydrogenase (Sigma-Aldrich, USA). After incubating the assay mixture without ATP at 30 °C for 5 min, the background activity of each sample was determined by recording the absorbance at 340 nm for 2 min. The obtained background activity was later subtracted from the volumetric activity for precise calculation of the specific GK activity. The reaction was initiated by the addition of 10 µL of ATP (100 mM) and monitored by measuring the absorbance at 340 nm for 5 min. Assays were performed routinely at room temperature in plastic 1.5-mL cuvettes (Bio-Rad, USA). An extinction coefficient of 6.22 mM^−1^ cm^−1^ was used for the calculation of the specific GK activity. One unit (U) is defined as the amount of enzyme required to consume 1 µmol NADH per minute. The protein concentration in the cell-free extracts was determined following the method described by Bradford [37], using bovine serum albumin A 3350 (Sigma-Aldrich, USA) as the standard.

## Results

### Adaptive laboratory evolution of the strain CEN.PK113-1A and characterization of the evolved isolates

As cells of the strains CEN.PK113-1A and CEN.PK113-7D did not grow at all in the synthetic glycerol medium used in this study, they were pre-grown in the same medium supplemented with a ready-to-use mixture of amino acids and nucleic bases (CSM). This medium supplement has been previously demonstrated to allow CEN.PK113-1A to grow on glycerol [20]. For the ALE experiments, two different regimes were used. In the first setup, the concentration of CSM was gradually reduced by 10% in each new batch cultivation, i.e., CSM supplementation was omitted starting from the tenth subcultivation. In the second setup, the pre-grown cells were directly transferred to synthetic glycerol medium without any further supplementation with CSM. In the first ALE setup, growth on glycerol of strain CEN.PK113-1A increased during the first 20 subcultivations (approx. 60 generations) up to a µ_max_ of 0.119 h^−1^ when aliquots were tested in medium without CSM (data not shown). As no further increase in the growth rate was observed during subsequent 10 subcultivations (approx. 40 generations), single-colony isolates were selected from a glycerol stock of the evolved culture taken after the twentieth subcultivation. Three single-colony isolates grown on solid synthetic glycerol medium were selected and characterized in detail in liquid synthetic glycerol medium (data not shown). As all of the isolates showed very similar growth behavior on glycerol, just one of them was selected for further study (referred to as PW-1).

In the second ALE setup, the µ_max_ of strain CEN.PK113-1A reached 0.040 h^−1^ after 6 subcultivations (approx. 20 generations) and further increased to 0.127 h^−1^ after 10 additional subcultivations (approx. 35 generations) (data not shown). Single-colony isolates were characterized as described above, and one isolate with representative growth performance was selected for further study (referred to as PW-2) (data not shown).

**Fig. 1:**
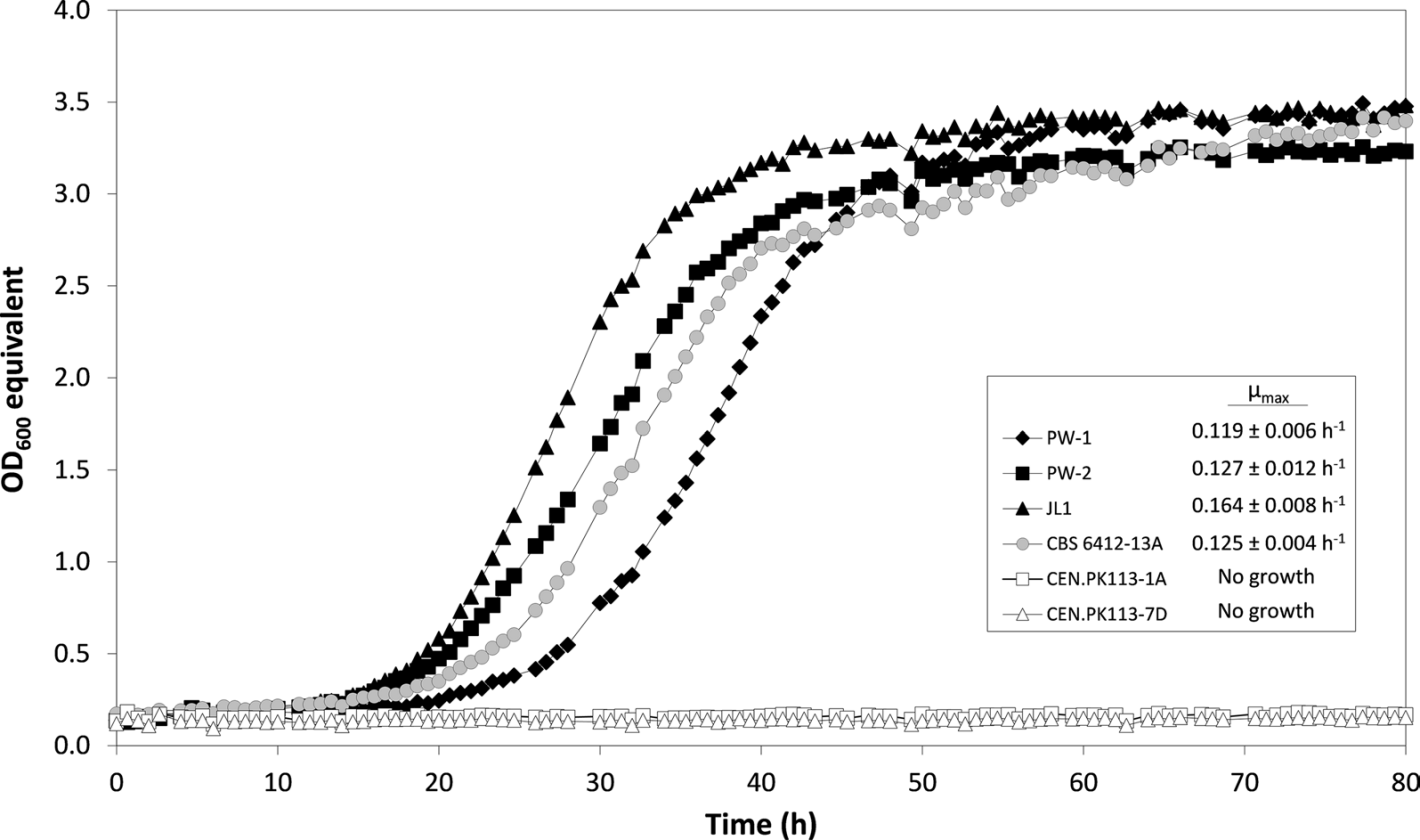
Growth performance of the evolved CEN.PK113 derivatives JL1, PW-1, and PW-2, and the wild-type strain CBS 6412-13A in synthetic glycerol medium. The unevolved strains CEN.PK113-1A and CEN.PK113-7D showing no growth on glycerol at all were included in the experiment as negative controls. All evolved strains were subjected to five serial transfers in synthetic glucose medium before quantifying the growth in synthetic glycerol medium. Data from one representative experiment out of three independent biological replicates are shown. Average values and standard deviations of µ_max_ are shown in the *box*

In a next step, the glycerol growth performances of the two evolved strains PW-1 and PW-2 were compared with that of the previously evolved CEN.PK derivative JL1 [5]. In order to minimize the effect of physiological adaptations of the three evolved strains that could have happened during the long-term cultivation in glycerol throughout the ALE experiment, all strains were first subjected to 5 subcultivations (approx. 25 generations) in liquid synthetic glucose medium. Afterward, the strains were analyzed for growth performance in synthetic glycerol medium using the Growth Profiler. For comparison, the wild-type strain CBS 6412-13A was also included in this experiment. As shown in Fig. 1, the strain JL1 exerted significantly better growth on glycerol compared to the strains PW-1 and PW-2 and the wild-type strain CBS 6412-13A. Therefore, we first focused on further analyzing strain JL1 in order to identify the crucial mutations underlying the strain’s improved growth on glycerol.

### The major part of its glycerol growth phenotype is determined by only two mutations

**Fig. 2:**
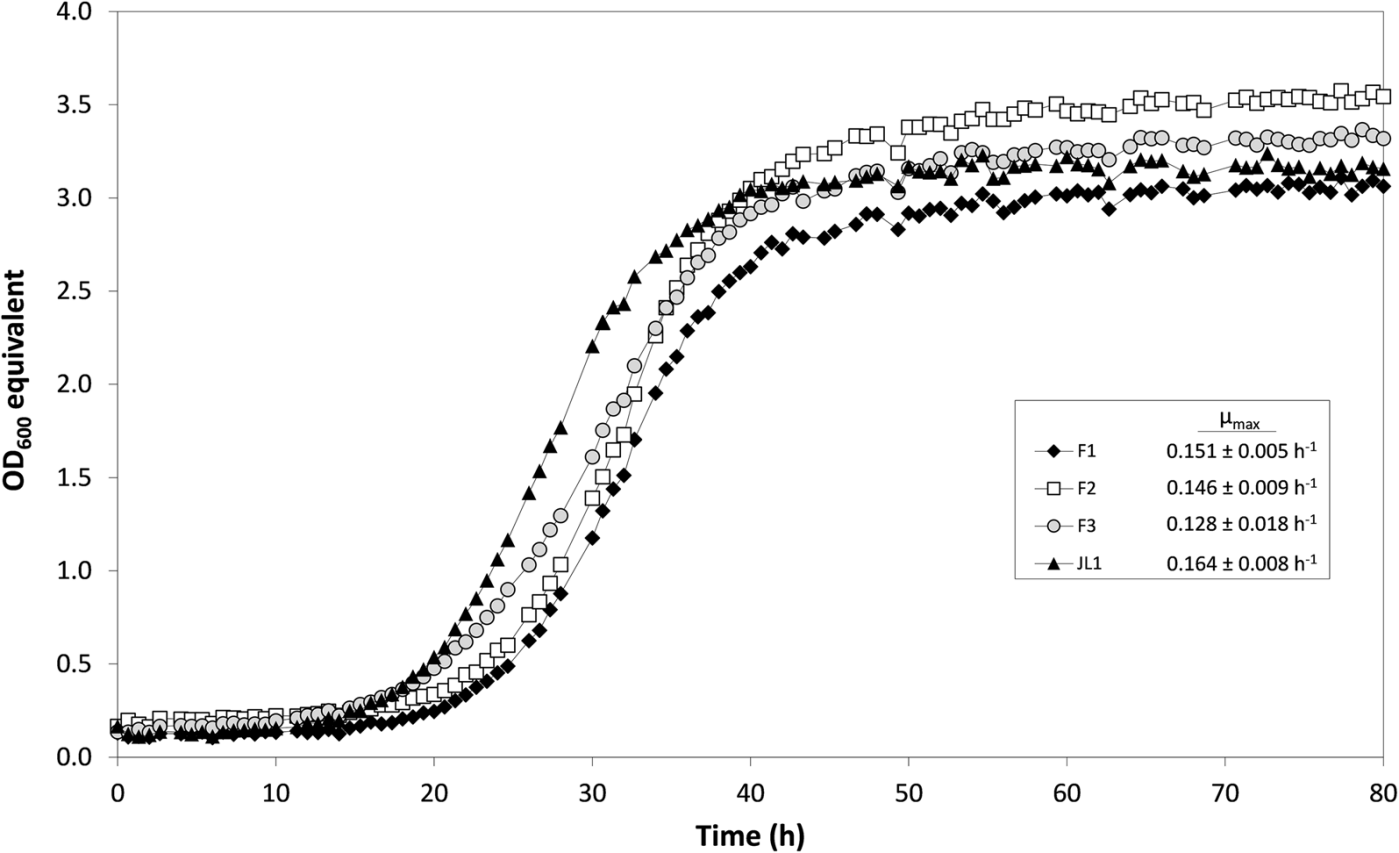
Growth performances of the selected segregants from each generation of backcrossing. One segregant of the cross between strains JL1 and CEN.PK113-7D, exerting the best growth performance on glycerol, was selected and denominated F1. This strain was backcrossed with strain CEN.PK113-7D for two successive rounds of backcrosses, yielding strains F2 and F3 both selected for the highest growth rate on glycerol among the respective progeny. Strain F3 was sent for whole-genome resequencing for further analysis. Data from one representative experiment out of three independent biological replicates are shown. Average values and standard deviations of µ_max_ are shown in the *box*

Before considering whole-genome resequencing of strain JL1 to identify the relevant mutations, we first reduced the number of mutations in the evolved strain by backcrossing the latter with its ancestral (unevolved) strain. We therefore switched the mating type of strain JL1 and then crossed the strain with strain CEN.PK113-7D. After sporulating the diploid hybrid strain, 28 haploid segregants were screened for growth in synthetic glycerol medium and the best performing strain (referred to as ‘F1’ in Fig. 2) was backcrossed with strain CEN.PK113-7D for two further rounds. Theoretically, 87.5% of the mutations originating from strain JL1 should be eliminated after three rounds of backcrossing. Out of the segregants of the F3 generation screened for growth on glycerol, one isolate (referred to here as ‘F3’) was selected for further study, showing a µ_max_ of 0.128 h^−1^ and lag phase of 15 h (Fig. 2). The genomic DNA (gDNA) of this strain together with the gDNA of the parental strains JL1 and CEN.PK113-7D was afterward sent for whole-genome resequencing.

**Fig. 3:**
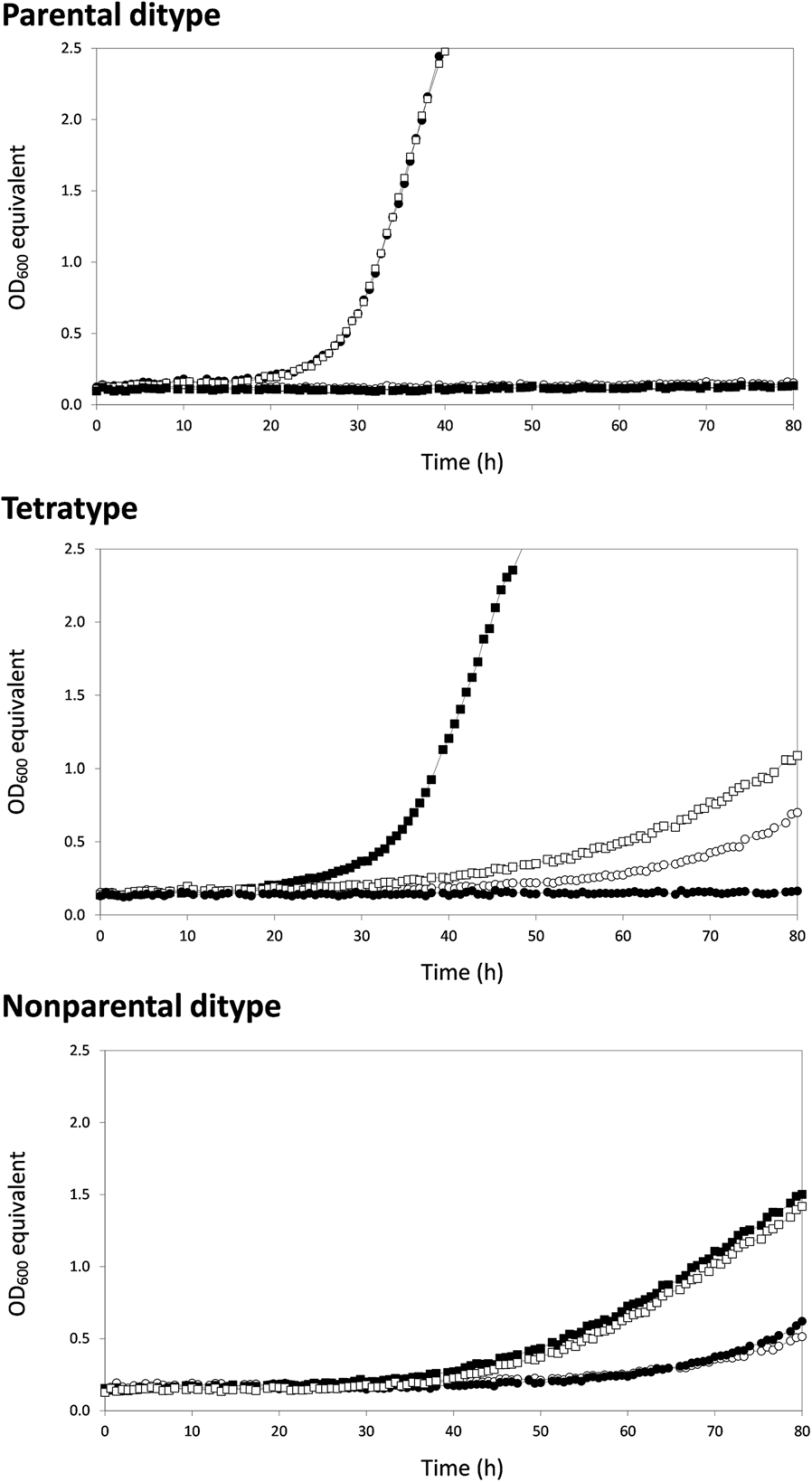
Exemplary representation of the glycerol growth performances for each tetrad pattern, i.e., Parental ditype, Tetratype, and Non-parental ditype. Segregants of each representative tetrad were obtained from the backcrossing experiment of strains JL1 and CEN.PK113-7D and individually assayed for their glycerol growth performances in liquid synthetic glycerol medium

In the course of backcrossing, a number of asci were analyzed with regard to the glycerol growth performance of the respective four individual segregants. Our data show that 16 out of the 20 analyzed tetrads corresponded to one of the three tetrad patterns described by Perkins [38], which are observed when two mutations are underlying a phenotype of interest. In particular, four tetrads of parental ditype (PD), eleven of tetratype (TT), and one of non-parental ditype (NPD) were identified. Figure 3 exemplarily shows the results for one tetrad representing each tetrad type. Although the number of screened tetrads was relatively small, the results strongly suggest that two major non-linked determinants are associated to the improved glycerol growth phenotype of the evolved strain JL1.

### Whole-genome resequencing analysis revealed two candidate gene variants relevant for the glycerol growth phenotype

Bioinformatic analysis of whole-genome resequencing data from the strains JL1 and F3 and the parental unevolved strain CEN.PK113-7D provided a raw dataset of 34,978 variants compared to the reference genome of the laboratory strain S288C (see methods for details). The high average coverage at which the samples were sequenced (over 40× for the three strains) allowed stringent filters to be applied on the raw dataset, removing unreliable variants between the three strains and S288C and obtaining a much smaller dataset of 5463 variants polymorphic within the three strains. About 94% of the variants in both the raw and the filtered dataset were biallelic single-nucleotide polymorphisms (SNPs). Manual inspection of the variants within the filtered dataset revealed a large number of heterozygous genotype calls that are generally unexpected for haploid strains. Because these heterozygous genotypes could only be the product of differences between repetitive elements or genotype errors [31, 39, 40], we further removed variants in which at least one sample showed a heterozygous genotype and variants in which copy number variation was predicted for the three samples. Only 10 variants (7 SNPs and 3 Indels) remained after applying these filters (Table 3).

**Table 3:** Filtered variant metrics for strains CEN.PK113-7D, JL1, and F3 using strain S288C as a reference genome

Chr.	Position	Reference (S288C)	CEN.PK113-7D	JL1	F3	Status	ORF	
VII	978,157	C	C	T	C	Lost in backcross	*LCS2*	Missense
VIII	38,156	G	G	A	A	Segregating	*GUT1*	Missense
IX	257,480	CAAAA	CAAAAAA	AAAAA	CAAAAAA	Lost in backcross	*RPL34B*	Upstream (+1000)
IX	287,261	C	A	C	A	Lost in backcross	*CST6*	Missense
XII	189,434	G	T	C	C	Segregating	*UBR2*	Nonsense
XII	829,429	ATGTAT	ATGTAT	AT	ATGTAT	Lost in backcross	*ORM2*	Downstream (-300)
XIII	758,921	GTATGT	GTATGT	GT	GTATGT	Lost in backcross	*FAA4*	Upstream (+1000)
XV	397,664	T	C	T	C	Lost in backcross	*YOR034C*-*A*	Missense
XVI	339,778	A	A	C	A	Lost in backcross	*CAR1*	Upstream (+1000)
M	2110	C	G	C	G	Lost in backcross	Intergenic	

Furthermore, 8 out of these 10 variants showed the expected genotype configuration for a mutation that was acquired by strain JL1 but lost in F3 due to the backcrossing with strain CEN.PK113-7D. The only two variants that were still present in strain F3 were located in the coding sequences of *GUT1* and *UBR2,* respectively. This observation is in accordance with the growth patterns of segregants within tetrads obtained during backcrossing (Fig. 3), where two mutations were predicted to be mainly relevant for the glycerol growth phenotype.

The first SNP resulted in a missense mutation from serine to phenylalanine at position 118 in the Gut1 protein (Table 4). The unevolved strain CEN.PK113-7D and the reference strain S288C, which cannot grow on synthetic glycerol medium at all [20], show a cytosine at position 353 in the coding sequence, whereas strains JL1 and F3 show an alternative nucleotide of thymine. The second SNP is located in the coding sequence of *UBR2* at position 3848 with a cytosine in the reference strain S288C but an adenine or guanine in the strains CEN.PK113-7D and JL1 (as well as in strain F3), respectively. Compared to the reference sequence of strain S288C, the SNP present in the strain CEN.PK113-7D changed the serine codon into a stop codon at position 1283 of the protein and hence led to an Ubr2 protein that is truncated by 590 amino acids at the C-terminus. In contrast, the nucleotide change identified within the same serine codon in the strain JL1 (and in strain F3) generated a tryptophan codon, but it resulted in a *UBR2* gene product that has the same total length as in strain S288C, i.e., 1872 amino acids (Table 4). All mutations were confirmed by traditional Sanger sequencing, validating that no mutation was present in either the promoter or terminator regions of the two alleles.

**Table 4:** List of all mutations in the *GUT1* and *UBR2* genes from the evolved strains and strain CBS 6412-13A

Position	*GUT1*						*UBR2*			
	353		1588		1597		3847		3848	
	Nucleotide	Amino acid	Nucleotide	Amino acid	Nucleotide	Amino acid	Nucleotide	Amino acid	Nucleotide	Amino acid
CEN.PK113-7D	C	Ser	G	Ala	G	Ala	A	STOP	T	STOP
JL1	T	Phe							G	Trp
PW-1					A	Thr	C	Gln		
PW-2			A	Thr			C	Gln		
CBS 6412-13A	Multiple [20]						Multiple [22]			

The positions are based on the genomic sequence of CEN.PK113-7D

### Allele swapping in strain CEN.PK113-7D verified the importance of the identified UBR2 and GUT1 mutations for the glycerol growth phenotype of the evolved strains

**Fig. 4:**
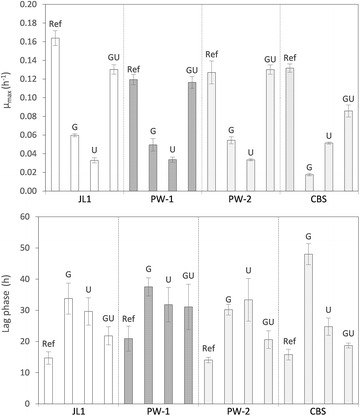
Reverse engineering of the strain CEN.PK113-7D for improved glycerol growth performance by allelic replacement of the GUT1 (G) and UBR2 (U) allele. The replacement cassettes obtained from the evolved strains JL1, PW-1, PW-2, and the wild-type isolate CBS 6412-13A (CBS) comprise the ORF and at least 1000 bp upstream and 300 bp downstream of the start and stop codon, respectively. The growth performance on glycerol of the reverse-engineered strains is presented by showing the growth rates and lag phases and compared to the growth performance of the respective reference strain (Ref) from where the replacement cassettes were obtained. Mean values and standard deviations were obtained from at least three biological replicates

After identifying the mutations in *UBR2*_JL1_ and *GUT1*_JL1_, allelic replacements were performed in the genetic background of the non-evolved CEN.PK113-7D strain in order to check the quantitative impact of the mutations on the glycerol growth phenotype. The respective allele was first deleted in strain CEN.PK113-7D and subsequently replaced by the corresponding allele from strain JL1. The whole procedure resulted in a seamless allele replacement, meaning that no foreign sequences or genetically selectable markers were left behind in the genomes of the reverse-engineered CEN.PK117-7D derivatives. In addition to using the mutant alleles from JL1, the same allele replacements were also conducted in the CEN.PK113-7D strain background with the respective *UBR2* and *GUT1* alleles originating from the above-mentioned strain CBS 6412-13A. Moreover, the respective alleles from the evolved strains PW-1 and PW-2 were also tested in the same genetic background due to the existing probability that the same two genes might have been affected during the ALE experiments performed in the current study. Maximum specific growth rates and lag phases for all reverse-engineered CEN.PK113-7D derivatives are shown in Fig. 4.

With regard to the JL1 mutations, one can see from Fig. 4 that the individual allele replacements with *GUT1*_JL1_ and *UBR2*_JL1_ allowed the strain CEN.PK113-7D to grow on synthetic glycerol medium with a µ_max_ of 0.060 and 0.033 h^−1^, respectively. When both genes were replaced by the respective JL1 alleles (*GUT1*_JL1_ and *UBR2*_JL1_), the resulting CEN.PK113-7D derivative exerted a µ_max_ of 0.130 h^−1^, which was higher than the sum of the µ_max_ obtained by the individual replacements of the two alleles (Fig. 4).

It can be recognized that the strain CEN.PK113-7D *UBR2*_CBS_ grows slightly better on glycerol as compared to the reverse-engineered CEN.PK strains with *UBR2* replaced by the evolved strains JL1, PW1, and PW2 (with regard to µ_max_ as well as lag phase) (Fig. 4). The opposite was observed for strains with the sole replacement of *GUT1* allele, for which the strain CEN.PK113-7D *GUT1*_CBS_ showed a lower µ_max_ and a longer lag phase compared to those of strains CEN.PK113-7D *GUT1*_JL1_, CEN.PK113-7D *GUT1*_PW-1_, and CEN.PK113-7D *GUT1*_PW-2_ (Fig. 4). The superior effect of the *GUT1*_JL1_ compared to *GUT1*_CBS_ can also be recognized in the respective strains where both alleles were combined (strain CEN.PK113-7D *GUT1*_JL1_ *UBR2*_JL1_ compared to strain CEN.PK113-7D *GUT1*_CBS_ *UBR2*_CBS_). Moreover, the replacement of *GUT1*_CBS_ by *GUT1*_JL1_ in the previously constructed strain CEN.PK113-1A *GUT1*_CBS_ *UBR2*_CBS_ [22] significantly increased the strain’s µ_max_ on glycerol from 0.08 to 0.14 h^−1^ (data not shown). Remarkably, the latter growth rate is even higher than the one of the wild-type strain CBS 6412-13A.

When the *UBR2* and *GUT1* alleles from strains PW-1 and PW-2 were replaced into the CEN.PK113-7D background, the effects on glycerol growth performance were relatively similar to those obtained after replacing the JL1 alleles (Fig. 4). Notably, the combination of the two allele replacements (with both PW-1 and PW-2 alleles) resulted in the same µ_max_ as the respective original evolved strains PW-1 and PW-2, respectively.

### The single-point mutations identified in the *UBR2* and *GUT1* alleles from the evolved strains PW-1 and PW-2 are different from those found in strain JL1

After the crucial role of the *UBR2* and *GUT1* alleles from strains PW-1 and PW-2 for their glycerol growth phenotype was confirmed, the respective alleles including the coding sequence and at least 1000 bp upstream and 300 bp downstream of the start and stop codon, respectively, were subjected to Sanger sequencing. The mutations found are shown in Table 4, alongside with the mutations found in the respective alleles of strain JL1. Data revealed that *UBR2*_PW-1_ and *UBR2*_PW-2_ are identical carrying one mutation in the coding sequences when compared to the coding sequence of *UBR2*_CEN.PK113-7D_. Similar to the mutation identified in the evolved JL1 strain, the mutation converted the stop codon present in the *UBR2*_CEN.PK113-7D_ allele into a glutamine (in contrast to a tryptophan in strain JL1).

The sequencing results for the *GUT1* alleles of the evolved strains PW-1 and PW-2 revealed a single (but different) SNP in the two coding sequences, and mutations are different from the single-point mutation identified in strain JL1. Both of the identified SNPs in strains PW-1 and PW-2 resulted in a missense mutation changing an alanine to a threonine at positions 533 and 530 in the Gut1 proteins, respectively. This is in contrast to the SNP detected in *GUT1*_JL1_ resulting in an exchange of serine to phenylalanine at position 118 in the corresponding protein (Table 4).

## Discussion

The goal of this study was to identify the crucial mutations enabling CEN.PK derivatives to utilize glycerol after adaptive laboratory evolution. Ochoa-Estopier et al. [5] generated a respective strain (JL1) that showed a µ_max_ on glycerol of 0.20 h^−1^ when analyzed directly after the ALE experiment, i.e., without any intermediate subcultivation in glucose-containing medium. The same strain only exerted a µ_max_ on glycerol of 0.16 h^−1^ in the current study after it was subjected to five subcultivations in glucose-containing medium. This discrepancy could have resulted from the use of slightly different growth conditions and media and/or the possibility that non-genetic adaptations might have appeared during the evolution, which contributed to the higher growth rate directly measured after the ALE experiment. One example of a non-genetic adaptation can be due to an increased mitochondrial activity resulting from a fully respiratory mode of carbon catabolism during growth on glycerol [41]. Another example has been suggested by Brickner [42] that yeast cells have cellular and molecular mechanisms to mark and promote previously expressed genes, a phenomenon the author coined “adaptive transcriptional memory” [42]. Certain carbon utilization-related genes are found relocalized to the nuclear periphery upon the shift of carbon source and retained stably for several generations. Such peripheral localization allows for a more robust reactivation of the genes and confers epigenetic memory of the previous transcriptional state [42, 43]. In the current study, the fact that among 28 segregants tested after backcrossing of strain JL1, no single segregant could be identified exerting the same µ_max_ as the parental strain JL1 supports the assumption of non-genetic adaptations occurred in the evolved strain JL1. Moreover, the growth rate of the best performing segregant obtained from each backcross gradually decreased from generation to generation (Fig. 2), eventually leading to a µ_max_ of 0.128 h^−1^ in the strain F3. This growth rate is comparable to the one of the reverse-engineered strain CEN.PK113-7D *GUT1*_JL1_ *UBR2*_JL1_ (0.130 h^−1^). In spite of these indications, we cannot completely exclude that this result is due to several minor advantageous mutations concertedly contributing to the higher growth rate of strain JL1 that were gradually lost during the backcrossing.

In the course of this work, the two crucial mutations in strain JL1 could be allocated to the genes *UBR2* and *GUT1*. It should be noted that one of the two crucial mutations could only be identified when analyzing the raw sequencing data against the sequence of the well-known *S.* *cerevisiae* strain S288C as a reference genome. In fact, only the point mutation within the *UBR2* gene was identified when performing the complete analysis of the raw sequencing data using the published assembly of the parental CEN.PK113-7D strain [32] as a reference. As the presence of a second crucial mutation was predicted with high confidence by the backcrossing results, we searched for this mutation in a new data analysis using the S288C reference sequence with a higher sequencing depth than the one from CEN.PK113-7D. It turned out that the region where the *GUT1* SNP is located was not sequenced in the CEN.PK113-7D assembly. It can be concluded that the quality of the reference sequence used was eventually more important for the quality of the analysis than the level of identity between the reference genome and the genome of the analyzed strain.

It was surprising to find that the same genes (*UBR2* and *GUT1*) were mutated in the three strains obtained in the independent ALE experiments for growth on glycerol (i.e., in the evolved strains JL1, PW-1, and PW-2). Several previously published adaptive evolution approaches of *S.* *cerevisiae* (such as for acetic acid and n-butanol tolerance) have shown that strains evolved in parallel experiments can have mutations affecting different sets of genes [44, 45]. Therefore, the results of the current study strongly suggest that *UBR2* and *GUT1* are absolutely crucial in the establishment of glycerol utilization capability in CEN.PK strains. This is also supported by the above-mentioned fact that the genes *UBR2* and *GUT1* have also been identified as targets to establish glycerol utilization in the strain CEN.PK113-1A when using a completely different approach [20, 22]. In the latter studies, we sought to identify the genetic basis of the difference in glycerol utilization capability between the *S.* *cerevisiae* wild-type isolate CBS 6412-13A (µ_max_ of 0.13 h^−1^) and the strain CEN.PK113-1A (no growth at all) by applying the method of pooled-segregant whole-genome resequencing [46]. The alleles of *UBR2*, *GUT1*, and *SSK1* were identified to be the genetic determinants of the glycerol growth phenotype of strain CBS 6412-13A. Interestingly, no mutation in *SSK1* was found in the genomes of the evolved strains (neither in the coding sequence nor in the promoter), and thus cannot be responsible for the above-mentioned difference in glycerol growth performance between strain JL1 and the reverse-engineered strain CEN.PK113-7D *GUT1*_JL1_ *UBR2*_JL1_.

Diversity, either natural or artificial, can both guide strain improvement via reverse engineering [47]. Both ALE experiments and the analysis of evolutionary distant strains by the pooled-segregant whole-genome resequencing approach have their pros and cons. Although ALE is only applicable when a selectable phenotype is in demand, two advantages of this method become obvious when comparing the current study with our previous genetic mapping approach [22]. Apart from being much less time-consuming, ALE has a higher potential to directly yield the causative mutations within crucial genes. To illustrate this, one has to note that 18 SNPs in the coding sequence and 10 SNPs within the respective promoter and terminator regions of *UBR2* were identified when comparing the strains CBS 6412-13A and CEN.PK113-1A [22]. In contrast, only a single-point mutation per allele (including the coding sequence as well as the promoter and terminator regions) was found in all evolved strains. In general, we only expected about 1 to 10 genome-wide mutations in the strain JL1 compared to strain CEN.PK113-7D. The reason is that Ochoa-Estopier et al. [5] applied only about 50 generations in their ALE experiment and the natural mutation rate in *S.* *cerevisiae* strains ranges from 10^−9^ to 10^−10^ mutations per base pair per generation [48, 49].

In the study of Swinnen et al. [22], the respective *UBR2*_CBS_ allele had the strongest impact on glycerol growth performance in strain CEN.PK113-1A, and the crucial SNP was a change of a premature stop codon into an amino acid encoding codon resulting in a longer Ubr2 protein. The same modification was found in the current study in all three independently evolved strains. It was therefore intriguing to check whether the sole presence of a full-length *UBR2* gene sequence correlates with the ability of *S. cerevisiae* strains to grow on glycerol. However, alignment of the *UBR2*_CEN_ allele against the corresponding alleles from other *S.* *cerevisiae* strains available on the SGD website (www.yeastgenome.org/) unveiled that the truncated *UBR2* allele is a specific characteristic of the CEN.PK family, and a full-length allele is not the sole criterion for glycerol utilization ability. In fact, the genomes of the well-known strains S288C, W303, and Ethanol Red encode for a full-length Ubr2 protein, but these strains still cannot grow at all in synthetic glycerol medium [20]. Moreover, the replacement of the *UBR2* allele in strains W303 and S288 by the *UBR2*_CBS_ allele did not establish growth on glycerol (unpublished data).

As *GUT1* encodes for glycerol kinase (GK), the first enzyme in the glycerol catabolic pathway of *S.* *cerevisiae* [50], it seems obvious that a higher in vivo activity of this enzyme could affect the efficiency of glycerol utilization. The respective mutation in the *GUT1* coding sequence might have resulted in modified regulatory properties of the enzyme or a different V_max_, e.g., by changes in posttranslational modifications or in a higher protein abundance caused by increased mRNA stability, translational efficiency, or protein stability. To test if a simple increase in Gut1 abundance is able to establish growth in synthetic glycerol medium, an additional copy of *GUT1* (driven by the strong *TEF1* promoter) was integrated in the genome of the wild-type strain CEN.PK113-7D. This, however, did not result in a detectable improvement of the growth on glycerol (data not shown).

Protein abundance can also be checked in vitro by measuring specific GK activities in crude cell extracts. As the strain CEN.PK113-7D with the wild-type *GUT1* allele cannot grow at all in synthetic glycerol medium, we used two compromises in order to compare its specific GK activity with that of the evolved strain JL1, and the reverse-engineered strain CEN.PK113-1A *GUT1*_JL1_ *UBR2*_JL1_. In the first approach, the specific GK activities of glucose-pre-grown cells were recorded at 1 and 4 h after shifting them from synthetic glucose to glycerol medium. In the second approach, CSM was added to the synthetic glycerol medium allowing the wild-type CEN.PK113-7D strain to grow [20] and to analyze the specific GK activities of all three strains during exponential growth on glycerol. As shown in the Additional file 1: Figure S1, we could not detect any significant differences regarding the respective specific GK activities between the wild-type strain and the reverse-engineered strain regardless the experimental setup used. Hence, it can be concluded that the identified mutation in *GUT1* does not seem to affect the abundance of the gene product in the cell, but rather might influence its regulatory properties causing an increased in vivo flux from glycerol to glycerol 3-phosphate. To experimentally verify this hypothesis, studies regarding enzyme kinetics have to be performed with purified wild-type and mutated Gut1 protein.

In contrast to the Gut1 protein, no direct involvement of Ubr2 in glycerol transport or catabolism is known so far. In general, Ubr2 is an E3 ubiquitin ligase, which usually acts together with a ubiquitin activating enzyme (E1) and a ubiquitin conjugation enzyme (E2) in the so-called ubiquitin proteasome system (UPS) which is a key mechanism for the spatiotemporal control of metabolic enzymes or disposal of misfolded and damaged proteins [51, 52]. In the UPS, the 76-amino acid ubiquitin is conjugated to lysine residues in target proteins through a series of reactions catalyzed by the above-mentioned E1–E2–E3 cascade of enzymes. The ubiquitin acts as a signal for the proteasome-mediated degradation of the respective target enzyme and this mechanism is particularly important for the acclimation of cells to changes in their environment. For a detailed review regarding the UPS system in yeast, the reader is referred to [51, 53]. It has been shown that Ubr2 has an overlapping function with its paralog, Ubr1, in the clearance of misfolded cytosolic proteins [54]. By far, the most prominent substrate identified for Ubr2 ligase is Rpn4, a major stress-induced transcription factor responsible for induction of proteasome subunits [55, 56]. The deletion of *UBR2* leads to Rpn4 stabilization, resulting in an elevated abundance of the proteasome that is suggested to positively correlate with longevity of the cells [57, 58].

It might be tempting to conjure that the truncated Ubr2 protein found in CEN.PK strains is a loss-of-function allele based on the result obtained from this study. Nonetheless, the result of Swinnen et al. [22] showed that the deletion of Ubr2 from the strain CEN.PK113-1A (i.e., the allele encoding the truncated Ubr2) in a CEN.PK113-1A × CBS 6412-13A hybrid background still resulted in an elongated lag phase and a reduced µ_max_ as compared to the hybrid strain CEN.PK113-1A × CBS 6412-13A [22]. Therefore, the exact mechanism by which the truncation of this allele affects the glycerol utilization in CEN.PK strains remains to be elucidated. Another obvious question is whether Ubr2 could be directly involved in the protein turnover of the three proteins predominantly involved in glycerol catabolism, that is Stl1, Gut1, and Gut2 [50]. However, these proteins do not belong to the so far identified substrates for ubiquitination according to the *S. cerevisiae* Ubiquitination Database (http://scud.kaist.ac.kr/). Moreover, there are experimental findings which might indicate that Ubr2 rather affects the glycerol utilization pathway downstream of dihydroxyacetone phosphate (DHAP). In fact, the replacement of *UBR2* also positively affected glycerol utilization in the CEN.PK 113-1A genetic background when the native glycerol catabolic pathway from glycerol to DHAP (‘glycerol 3-phosphate pathway’) was replaced by the ‘dihydroxyacetone phosphate’ pathway. Notably, the latter pathway is independent of Gut1 and Gut2 enzyme activities and glycerol is assumed to be predominantly taken up via a heterologous glycerol facilitator [59].

## Conclusions

The current work confirmed the importance of *UBR2* and *GUT1* as targets for establishing glycerol utilization in strains of the CEN.PK family. In addition, it shows that a growth rate on glycerol of 0.130 h^−1^ can be established in reverse-engineered CEN.PK strains by solely replacing two single amino acids within the coding sequence of Ubr2 and Gut1. Future work will focus on studying the molecular basis for the positive impact of the single-point mutations on the glycerol growth phenotype.

## Additional file

**Additional file 1: Figure S1.** Specific in vitro glycerol kinase (GK) activity in the wild-type strain CEN.PK113-7D, the evolved strain JL1 and the reverse-engineered strain CEN.PK113-7D *GUT1*_JL1_ *UBR2*_JL1_. First, GK activities were recorded 1 and 4 h after shifting the cells from glucose to glycerol. For this experiment, cells were precultivated in shake flasks in synthetic medium containing 2% (w/v) glucose. When the culture had reached exponential growth phase (OD_600_ of ~ 1.2), cells were washed once with synthetic medium containing 6% (v/v) glycerol before inoculating the washed cells in the latter medium adjusting OD_600_ to 1.0. The cultures were incubated at 30 °C and 200 rpm before measuring the GK activity. (*) Second, in order to allow the comparison of specific GK activities during exponential growth in glycerol medium, the medium was supplemented with CSM. This allowed the wild-type strain CEN.PK113-7D to also grow on glycerol. In fact, all three strains showed similar maximum specific growth rates (~0.12 h^−1^). Mean values and standard deviations for specific GK activity were obtained from three biological replicates.

## Abbreviations

ALE: adaptive laboratory evolution
ADP: adenosine diphosphate
ATP: adenosine triphosphate
*ble*^*r*^: phleomycin resistance
bp: base pair
CNV: copy number variation
CSM: complete supplement mixture
DHAP: dihydroxyacetone phosphate
DNA: deoxyribonucleic acid
gDNA: genomic DNA
GK: glycerol kinase
GQ: genotype quality
G-value: green value
Indel: small insertion and deletion
MAT: mating type
NADH: nicotinamide adenine dinucleotide
NPD: non-parental ditype
OD: optical density
ORF: open reading frame
PCR: polymerase chain reaction
PD: parental ditype
SNP: single-nucleotide polymorphism
TT: tetratype
µ_max_: maximum specific growth rate
UPS: ubiquitin proteasome system
V_max_: maximum velocity
YPD: yeast extract—peptone growth medium with dextrose
YPGal: yeast extract—peptone growth medium with galactose
