# Supplementary material for: The sole introduction of two single-point mutations establishes glycerol utilization in Saccharomyces cerevisiae CEN.PK derivatives
Source: Biotechnol Biofuels. 2017 Jan 3;10:10. doi: 10.1186/s13068-016-0696-6 (PMC5209837; doi:10.1186/s13068-016-0696-6)
Supplement: Supplementary file 1 — Additional file 1: Figure S1. Specific in vitro glycerol kinase (GK) activity in the wild-type strain CEN.PK113-7D, the evolved strain JL1 and the reverse-engineered strain CEN.PK113-7D GUT1 JL1 UBR2 JL1. First, GK activities were recorded 1 and 4 h after shifting the cells from glucose to glycerol. For this experiment, cells were precultivated in shake flasks in synthetic medium containing 2% (w/v) glucose. When the culture had reached exponential growth phase (OD600 of ~ 1.2), cells were washed once with synthetic medium containing 6% (v/v) glycerol before inoculating the washed cells in the latter medium adjusting OD600 to 1.0. The cultures were incubated at 30 °C and 200 rpm before measuring the GK activity. (*) Second, in order to allow the comparison of specific GK activities during exponential growth in glycerol medium, the medium was supplemented with CSM. This allowed the wild-type strain CEN.PK113-7D to also grow on glycerol. In fact, all three strains showed similar maximum specific growth rates (~0.12 h−1). Mean values and standard deviations for specific GK activity were obtained from three biological replicates. [file 13068_2016_696_MOESM1_ESM.docx]

Additional Figure 1

JL1

CEN.PK113-7D *GUT1*_JL1_ *UBR2*_JL1_

CEN.PK113-7D
